# Critical Evaluation of the Interaction of Reactive Oxygen and Nitrogen Species with Blood to Inform the Clinical Translation of Nonthermal Plasma Therapy

**DOI:** 10.1155/2020/9750206

**Published:** 2020-12-03

**Authors:** Abraham Lin, Eline Biscop, Colum Breen, Stephen J. Butler, Evelien Smits, Annemie Bogaerts

**Affiliations:** ^1^PLASMANT-Research Group, University of Antwerp, 2601 Antwerpen-Wilrijk, Belgium; ^2^Center for Oncological Research―Integrated Personalized & Precision Oncology Network (IPPON), University of Antwerp, 2601 Antwerpen-Wilrijk, Belgium; ^3^Department of Chemistry, Loughborough University, LE11 3TU Loughborough, UK; ^4^Center for Cell Therapy and Regenerative Medicine, Antwerp University Hospital, 2650 Antwerp-Edegem, Belgium

## Abstract

Non-thermal plasma (NTP), an ionized gas generated at ambient pressure and temperature, has been an emerging technology for medical applications. Through controlled delivery of reactive oxygen and nitrogen species (ROS/RNS), NTP can elicit hormetic cellular responses, thus stimulating broad therapeutic effects. To enable clinical translation of the promising preclinical research into NTP therapy, a deeper understanding of NTP interactions with clinical substrates is profoundly needed. Since NTP-generated ROS/RNS will inevitably interact with blood in several clinical contexts, understanding their stability in this system is crucial. In this study, two medically relevant NTP delivery modalities were used to assess the stability of NTP-generated ROS/RNS in three aqueous solutions with increasing organic complexities: phosphate-buffered saline (PBS), blood plasma (BP), and processed whole blood. NTP-generated RNS collectively (NO_2_^−^, ONOO^−^), H_2_O_2_, and ONOO^−^ exclusively were analyzed over time. We demonstrated that NTP-generated RNS and H_2_O_2_ were stable in PBS but scavenged by different components of the blood. While RNS remained stable in BP after initial scavenging effects, it was completely reduced in processed whole blood. On the other hand, H_2_O_2_ was completely scavenged in both liquids over time. Our previously developed luminescent probe europium(III) was used for precision measurement of ONOO^−^ concentration. NTP-generated ONOO^−^ was detected in all three liquids for up to at least 30 seconds, thus highlighting its therapeutic potential. Based on our results, we discussed the necessary considerations to choose the most optimal NTP modality for delivery of ROS/RNS to and via blood in the clinical context.

## 1. Background

**Figure 1:**
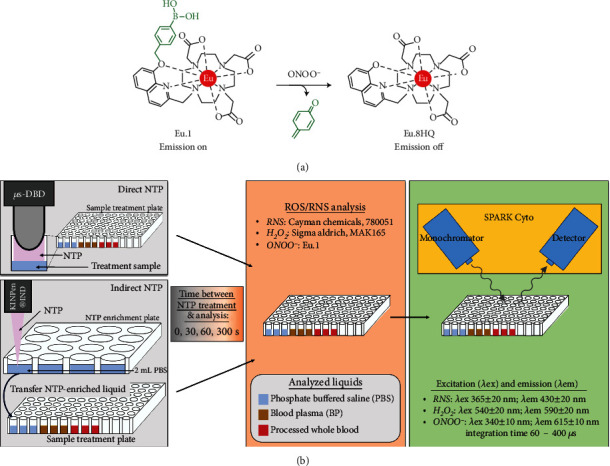
Experimental design to determine the stability of persistent ROS/RNS. (a) The mode of action of **Eu.1** for detecting ONOO^−^ is shown. (b) PBS (blue), BP (yellow), and processed whole blood (red) were analyzed following direct and indirect NTP treatment. Following treatment, the sample was either analyzed immediately (0 s) or after a delay (between 30 and 300 s) for RNS, H_2_O_2_, or ONOO^−^ (*μ*s-DBD: microsecond-pulsed dielectric barrier discharge; *λ*_ex_: excitation wavelength; *λ*_em_: emission wavelength).

In the past decade, non-thermal plasma (NTP) has been an emerging technology with diverse medical applications [1]. NTP is an ionized gas that can be generated at ambient pressure and temperature (25-40°C) and has been reported to enhance hemostasis, promote wound healing, and even kill cancerous cells [2–4]. This broad range of biological effects is a result of the reactive oxygen and nitrogen species (ROS/RNS) generated by NTP, including atomic species, free radicals, hydrogen peroxide, and nitrogen oxides, and the cellular phenomena, hormesis [1]. While low-dose exposure to ROS/RNS can promote regenerative effects, higher doses can induce cellular death. Through controlled delivery of ROS/RNS, the clinical benefits of NTP therapy to date have included neutralizing microorganisms, reducing chronic leg ulcers, resolution of dermatological diseases, and palliation for advanced head and neck cancer patients [5–10]. Furthermore, ongoing preclinical research indicates expanding application potential, which include stopping bleeding and mediating scar formation, alleviating burns, neuroregeneration, and tissue regeneration [4, 11–17]. This is in stark contrast to thermal plasma devices predominantly used for thermal ablation (≥60°C) [18, 19]. Two NTP delivery methods are currently considered most applicable for the clinic: direct NTP treatment and indirect NTP treatment [20]. In the direct setting, NTP is generated in direct contact with the target substrate (e.g., blood, wound, and tumor). This modality allows for the most efficient delivery of ROS/RNS but is currently limited to superficial areas that are accessible to the device. Indirect NTP treatment is one way to overcome this limitation. Here, NTP is used to enrich a solution with ROS/RNS, which can then be transferred to the target substrate. While this allows for treatment of areas deeper in the body, only persistent ROS/RNS (lifetimes ≥ *s*) may reach the target as short-lived ROS/RNS (lifetimes < *s*) may have off-target reactions or form more stable species during transport [21]. Therefore, investigation of ROS/RNS stability is a high priority for successful clinical translation of NTP technology. To date, experimental and *in silico* studies characterizing NTP-generated species in the liquid phase have mainly been performed in water or PBS [21–24], as this reduces the complexity of reactions with supplements and organic molecules found in other solutions (e.g., cell culture medium, blood). While this has led to significant in-depth insight into the fundamental physical and chemical NTP interactions with liquid, a major gap in current knowledge is on NTP interactions with patient material, as these substrates are far more complex than the models previously used. Since NTP-generated ROS/RNS will inevitably interact with blood in several clinical contexts, both deliberately (e.g., hemostasis) or inadvertently (e.g., cancer therapy), understanding their stability in this system is of major importance. In this study, we investigated the stability of NTP-generated ROS/RNS in blood as a first step toward elucidating NTP chemistry in clinical material. Both direct and indirect NTP delivery methods, previously used in our lab [25–27], were tested. We performed analysis of NTP-generated RNS collectively (nitrite, NO_2_^−^; peroxynitrite, ONOO^−^), hydrogen peroxide (H_2_O_2_), and ONOO^−^ over time in aqueous solutions with increasing organic complexities: phosphate-buffered saline (PBS), blood plasma (BP), and processed whole blood. Both RNS and H_2_O_2_, stable in PBS, were scavenged by different components of the blood. Our previously developed luminescent europium(III) probe, **Eu.1** (Figure 1(a)), [28] was also used for precision measurement of ONOO^−^ and revealed that NTP-generated ONOO^−^ was present in all three liquids, for at least 30 seconds. Based on our results, we discussed the scavenging capacity of different blood components as well as the necessary considerations to choose the most optimal NTP modality for the desired clinical application.

## 2. Methods

### 2.1. Aim and Experimental Design

The aim of this study was to evaluate the stability of NTP-generated persistent ROS/RNS in clinical substrates (e.g., blood plasma and processed whole blood) to inform on the optimal translation of NTP technology per application. Direct and indirect NTP applications were evaluated and three chemical analysis methods were used (Figure 1(b)). The concentration of ROS/RNS generated immediately in PBS (0 s) was used as the baseline concentration following NTP treatment. The delay between the end of NTP treatment and the chemical analysis was varied between 30 and 300 seconds to provide insight into ROS/RNS stability over time.

### 2.2. Collection of BP and Processed Whole Blood

This study was approved by the Ethics Committee of the University of Antwerp/Antwerp University Hospital (Antwerp, Belgium) under the reference number 19/13/160. Experiments were performed using blood samples from anonymous donors provided by the Blood Service of the Red Cross Flanders (Mechelen, Belgium). The BP was separated from the blood using the following protocol. 25 mL processed whole blood was carefully dispensed onto 15 *μ*L LymphoPrep (1114547, Alerne Technologies SA) and centrifuged for 20 minutes at 2100 rpm at room temperature in a swing-out rotor. After centrifugation, the blood components were separated into four distinct layers with BP at the top. This top layer of BP was then easily collected from the other parts of the blood. The term “processed whole blood” refers to buffy coats that were derived from healthy volunteers' peripheral blood (with citrate phosphate dextrose anticoagulant) donated the day before and stored overnight at room temperature (Red Cross Flanders).

### 2.3. NTP Treatment

We studied both direct and indirect NTP treatment. A single treatment parameter for each method was used based on comparable therapeutic effects from our past studies and published reports [25, 26]. For the direct treatment, we used a custom-built microsecond-pulsed dielectric barrier discharge (*μ*s-DBD) operated at atmospheric pressure in air (Megaimpulse Ltd, St. Petersbug, Russia). For these experiments, we used a 700 Hz pulse frequency and treated the samples for 10 seconds, 1 mm above the samples.

For the indirect treatment, we used the kINPen®IND non-thermal plasma jet (INP Greifswald/Neoplas tools GmbH, Greifswald, Germany). This is an atmospheric pressure argon NTP jet, commonly used for plasma medicine research [26, 27, 29]. We used the kINPen®IND to treat 2 mL phosphate-buffered saline (PBS) (pH 7.3) in a 12-well plate. A gap of 6 mm between the tip of the NTP source and the liquid, a gas flow rate of 1 slm, and a treatment time of 5 minutes were used. Following treatment, the NTP-enriched PBS was transferred to the aqueous solution (PBS, BP, or processed whole blood).

### 2.4. RNS Measurement

To measure RNS concentration in NTP-treated PBS, BP, and processed whole blood, a fluorometric assay was used. The Nitrate/Nitrite Fluorometric Assay kit (780051, Cayman Chemical) was performed according to the manufacturer's instructions. Briefly, for direct treatment, each well contained 10 *μ*L of NTP-treated sample. For the indirect treatment, 10 *μ*L of NTP-enriched PBS (pPBS) was transferred to each well containing 40 *μ*L of PBS, BP, or processed whole blood. The final volume for each sample, both for the direct and indirect NTP treatment, was adjusted to 100 *μ*L with assay buffer. 10 *μ*L of 2,3-diaminonaphthalene (DAN) reagent was added to each well, either immediately or 30, 60, or 300 seconds after treatment. After 10 minutes of incubation at room temperature, 20 *μ*L of sodium hydroxide (NaOH) was added to each well to enhance the detection of the fluorescent product, 1(H)-naphthotriazole. Immediately after adding NaOH, fluorescence was measured with the Tecan Spark Cyto using an excitation wavelength (*λ*_ex_) of 365 ± 20 nm, an emission wavelength (*λ*_em_) of 430 ± 20 nm, and a fixed gain (gain 55). The selectivity of the Nitrate/Nitrite Fluorometric Assay Kit used in this study was also evaluated (Supporting Figure S1).

### 2.5. H_2_O_2_ Measurement

To measure H_2_O_2_ concentrations in NTP-treated PBS, BP, and processed whole blood, we performed a fluorometric assay (MAK165, Merck) according to the manufacturer's instructions. In short, for analysis of direct NTP treatment, each well contained 25 *μ*L of NTP-treated PBS or 50 *μ*L of NTP-treated BP or processed whole blood. The volume of each sample was adjusted with assay buffer to obtain a final volume of 50 *μ*L. In the case of indirect NTP treatment, each well contained either 2.5 *μ*L of pPBS adjusted with 47.5 *μ*L of PBS, 25 *μ*L of BP, or 25 *μ*L of processed whole blood. Different dilutions were used to stay within the calibration range. 50 *μ*L of master mix (4.75 mL Assay Buffer+50 *μ*L Red Peroxidase Substrate+200 *μ*L 20 units/mL Peroxidase) was then added to each well. After 30 minutes of incubation, fluorescence was measured with the Tecan Spark Cyto, (*λ*ex = 540 ± 20 nm, *λ*em of 590 ± 20 nm, fixed gain 34).

### 2.6. ONOO^−^ Measurement

Here, we used a new luminescent europium(III) probe (**Eu.1**, Figure 1(a)) which we developed previously, for detection of ONOO^−^*in vitro* and in living cells [28]. For initial time points, 100 *μ*L of **Eu.1** (100 *μ*M) in relevant media was treated with direct NTP and the emission intensity of **Eu.1** was measured immediately. For the subsequent time points, **Eu.1** (100 *μ*M) was added posttreatment with direct NTP after a certain amount of time (*t* = 30, 60, 120, and 300 seconds). Emission intensity was measured with a plate reader using time-resolved emission (*λ*_ex_ = 340 nm, *λ*_em_ = 615 ± 10 nm, int.time = 60–400 *μ*s). The concentration was determined by comparing the percentage change in emission intensity to the calibration curve (Supporting Figure S2).

### 2.7. Statistical Analysis

All statistical differences were analyzed using the linear mixed model with JMP Pro 13 (SAS software). The fixed effect was set to either the treatment or the time delay between treatment and analysis. When a significant difference was detected, the post hoc Dunnett's test was performed to calculate the adjusted *p* value compared to the control. For quantification of ROS/RNS species, NTP treatment at all time points was compared to the untreated. To determine the scavenging capacity of BP and processed whole blood, delayed analysis (30-300 seconds) of ROS/RNS concentration was compared to the initial (0 second). A *p* value of <0.05 was considered statistically significant. Data in all graphs are represented as mean ± standard error of the mean (SEM), the number of replicates are indicated in the legend, and all figures were prepared in GraphPad Prism (GraphPad Prism 7, GraphPad Prism Software, Inc.).

## 3. Results

### 3.1. NTP-Generated Persistent ROS/RNS Are Stable in PBS, but Scavenged Over Time in BP and Processed Whole Blood

**Figure 2:**
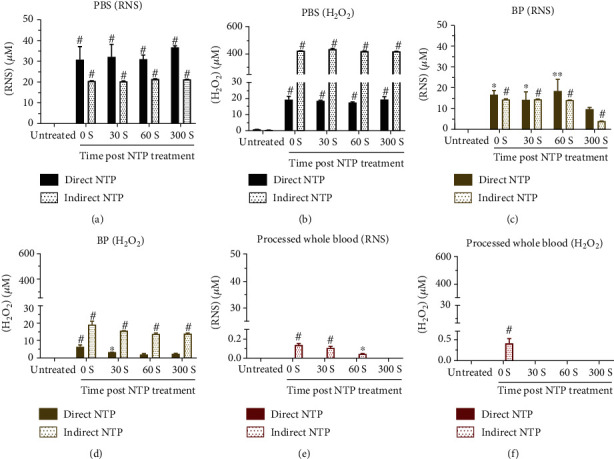
Quantification of persistent RNS and H_2_O_2_ in three aqueous solutions: PBS, blood plasma (BP), and processed whole blood. NTP was generated in direct contact with the target liquid (direct NTP) or used to enrich PBS, which was then transferred to the target liquid (indirect NTP). Following NTP treatment, RNS and H_2_O_2_ analysis was performed immediately (0 s) or after a delay of 30 to 300 seconds to assess the stability of these species in (a, b) PBS, (c, d) BP, and (e, f) processed whole blood. Data are represented as mean ± SEM. ROS/RNS concentration following NTP treatment at all time points was compared to untreated and statistical significance was determined using the generalized linear mixed model (*n* = 3). ^∗^*p* < 0.05; ^∗∗^*p* < 0.01; ^#^*p* < 0.001.

To determine the baseline concentrations of NTP-generated persistent ROS/RNS, direct and indirect NTP was used to treat PBS, using a DBD at atmospheric air and an argon kINPen jet, respectively. While direct NTP generated slightly higher concentrations of RNS compared to the indirect treatment (Figure 2(a)), indirect NTP produced over 20 times more H_2_O_2_ (Figure 2(b)). RNS and H_2_O_2_ analysis was performed immediately after NTP treatment (0 seconds) and delayed between 30 to 300 seconds after treatment. Both these species were stable in PBS up to at least 300 seconds. The pH of the PBS was also measured before and after NTP treatment and remained relatively unchanged following exposure to both direct (7.25 ± 0.01; mean ± SEM) or indirect (7.25 ± 0.01) NTP, compared to untreated (pH: 7.30 ± 0.01).

To examine the stability of NTP-generated ROS/RNS in a more complex physiological solution, we treated BP derived from healthy donors. BP accounts for approximately 55% of the volume of blood and consists primarily of water, dissolved electrolytes, proteins, and lipids. Both direct and indirect NTP were used to treat BP, which was collected and analyzed for RNS and H_2_O_2_ in the same manner as before. Interestingly, the RNS were immediately scavenged to around 15 *μ*M for both direct and indirect NTP treatment, despite direct treatment producing almost twice the amount of RNS in PBS (cf.  Figure 2(c) vs. Figure 2(a)). The remaining RNS were stable up to at least 60 seconds. On the other hand, the H_2_O_2_ concentration was immediately and significantly reduced compared with that in PBS and continued to decrease over time (Figure 2(d)).

Processed whole blood, which comprises red and white blood cells and platelets along with BP, was also treated and analyzed as the model for the most complex and complete physiological solution. Both RNS and H_2_O_2_ generated by direct NTP were immediately reduced to undetectable levels (Figures 2(e) and 2(f)). For indirect NTP, the RNS were immediately reduced below 0.2 *μ*M and were completely absent by 300 seconds, while H_2_O_2_ was only detectable immediately after treatment.

**Figure 3:**
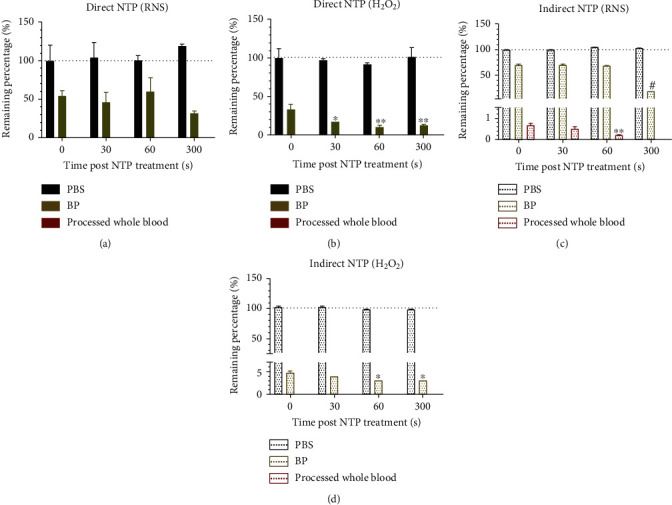
Scavenging capacity of BP and processed whole blood for NTP-generated ROS/RNS. RNS and H_2_O_2_ concentrations at different analysis time points were normalized to their baseline concentrations, measured immediately after treatment in PBS, to determine the remaining percentage following (a, b) direct NTP and (c, d) indirect NTP. Data are represented as mean ± SEM. The ROS/RNS concentration after NTP at time points 30-300 s was compared to the initial concentration (0 s) in the associated liquid. This comparison provides insight into the stability of the species over time in PBS, BP, and processed whole blood. The generalized linear mixed model was used to determine statistical significance (*n* = 3). ^∗^*p* < 0.05; ^∗∗^*p* < 0.01; ^#^*p* < 0.001.

While both ROS/RNS were stable in PBS, once introduced to the components of BP, their concentration was significantly diminished (Figure 3). In fact, the RNS concentration in BP was reduced to 50-75% of its starting concentration compared with that of PBS and was almost completely absent in processed whole blood (Figures 3(a) and 3(c)). On the other hand, immediate detection of H_2_O_2_ in BP displayed a remaining percentage of 33% for direct NTP (Figure 3(b)), and 4.5% for indirect NTP (Figure 3(d)) compared to baseline levels in NTP-treated PBS. H_2_O_2_ also decreased slightly over time in BP, which did not occur in PBS. Furthermore, at higher initial concentrations of H_2_O_2_ from indirect NTP treatment, H_2_O_2_ was still detectable in processed whole blood when immediately analyzed (Figure 2(f)), though the remaining percentage was ≤0.1% (Figure 3(d)). Therefore, while the cellular and platelet components may contribute to the H_2_O_2_ scavenging capacity of the blood, the BP components alone are potent scavengers.

### 3.2. NTP-Generated Peroxynitrite (ONOO^−^) Is Stable in PBS, BP, and Processed Whole Blood for up to at Least 30 Seconds

ONOO^–^ is a highly reactive RNS involved in cellular signaling and manipulating the ONOO^–^ concentration has been a strategy for therapy and management of several diseases (e.g., inflammatory disease, vascular diseases, and cancer) [30–32]. Detection of ONOO^–^ has been particularly challenging in the biomedical setting due to the limited selectivity and applicability of current assays and the slow response times of detection probes, notwithstanding some notable recent developments [33–35]. Here, we used a new luminescent europium(III) probe (**Eu.1**) to detect direct NTP-generated ONOO^–^ in PBS, BP, and processed whole blood. This probe, designed and synthesized by some of the authors, has been previously demonstrated to rapidly and selectively detect ONOO^–^ in a time-resolved manner, in aqueous buffers and human BP [28]. In the current study, the long luminescence lifetime of the Eu(III) probe has been utilized to eliminate any short-lived autofluorescence arising from biomolecules in the BP or processed whole blood, thereby providing high signal-to-noise and accurate measurements of ONOO^–^ concentration.

**Figure 4:**
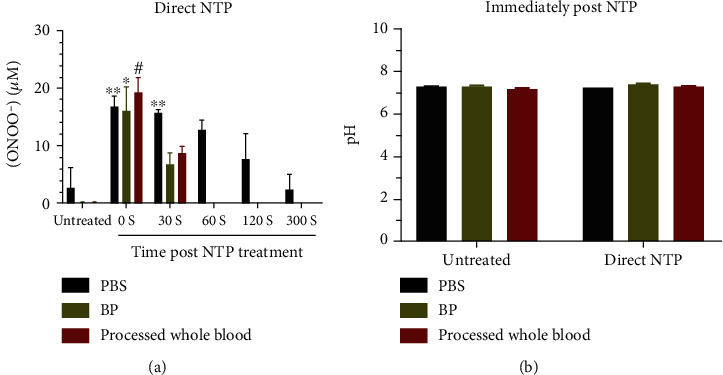
Analysis of NTP-generated ONOO^−^ stability in PBS, BP, and processed whole blood. (a) ONOO^−^ concentrations were analyzed by the addition of **Eu.1** (100 *μ*M) to the solution after a specific time post-NTP treatment. Data are represented as mean ± SEM. The ONOO^−^ concentration was compared to untreated in corresponding solutions, and statistical significance was determined using the generalized linear mixed model (*n* = 2‐10). ^∗^*p* < 0.05; ^∗∗^*p* < 0.01; #*p* < 0.001. (b) The pH of all aqueous solutions remained largely unchanged after direct NTP treatment (*n* = 3).

It is clear that ONOO^–^ is generated immediately following direct NTP treatment and the concentration is equivalent in all three aqueous solutions (Figure 4(a)). This indicates that direct treatment is initially generating a relatively constant concentration of ONOO^–^ (~17 *μ*M), regardless of the complexity of the medium. In PBS, the decay of ONOO^–^ occurs over 300 seconds and could be attributed to the reaction of ONOO^–^ to form more stable species such as nitrite (NO_2_^–^) and nitrate (NO_3_^–^) [36]. It is important to note that degradation of ONOO^–^ may not be solely and directly responsible for the formation of NO_2_^–^ and NO_3_^–^ and can result in significantly shorter-lived species (e.g., ONOOCO_2_^–^, NO) [22]. Interestingly, following a 30-second delay, ONOO^−^ in BP and processed whole blood was reduced to approximately the same amount, 7 ± 2 *μ*M and 9 ± 1 *μ*M, respectively, before becoming completely abrogated at higher time delays (≥60 seconds). This indicates that BP components play an important role in scavenging ONOO^–^, particularly NTP-generated ONOO^–^. As ONOO^–^ formation and stability are also highly dependent on the pH of the solution [37], we also evaluated the effect of NTP treatment on pH. Direct NTP did not significantly affect the pH in any of the aqueous solutions (<0.15 pH units) (Figure 4(b)). Taken together, this data strongly suggests that the decay of ONOO^–^ is due to reactions with other biomolecules/organic species in the blood, predominantly in the BP. This is not unexpected, given the potent nitrating and oxidizing nature of ONOO^–^ and the number of potential reactive partners in both processed whole blood and BP (e.g., amino acids, CO_2_, Fe^2+^) [36]. While the reaction of ONOO^–^ with biomolecules/organic species appears to be the predominant mechanism of decay, the interactions between other ROS/RNS cannot be entirely discounted.

## 4. Discussion

In this study, we tested the scavenging capacity of different components of blood, following treatment with two NTP modalities and using a range of complementary detection methods. Currently, a full understanding of how NTP-generated ROS/RNS interact with clinical substrates is lacking, and to our knowledge, this is the first report on the evaluation of NTP-generated ROS/RNS in blood.

Since RNS were reduced to a fixed concentration, despite the two NTP modalities producing different starting amounts (cf Figures 2(a) and 2(c)), this suggests that dissolved proteins (e.g., albumin, globulins, and fibrinogen) and lipids (e.g., fatty acids, cholesterol) in the BP only have a partial scavenging action. The additional cellular and platelet components of the processed whole blood further scavenged the RNS (Figures 3(a) and 3(c)). These data strongly suggest that the RNS are only partially reduced by the BP component of the blood. On the other hand, our results suggest that the BP contains major scavenging capacity for H_2_O_2_, as the remaining percentage of H_2_O_2_ was under 40% and 5% for direct and indirect treatment, respectively (Figures 3(b) and 3(d)). However, this is not to say that the cellular and platelet components of the blood do not contribute to scavenging of H_2_O_2_, as processed whole blood nearly quenched all H_2_O_2_ immediately (Figure 2(f)). This immediate scavenging effect of H_2_O_2_ is mostly due to the enzymatic machineries from the red blood cells, protecting hemoglobin from oxidative processes. It should be considered that the iron bound in the hemoglobin can be oxidized by H_2_O_2_ to ferryl hemoglobin (HbFe^4+^) [37]. With high concentrations of H_2_O_2_ (6.5 mM), this would decrease the amount of the oxygen-carrying form of hemoglobin, ferrous hemoglobin (HbFe^2+^O_2_), leading to toxic responses [37]. When produced in small amounts, HbFe^4+^ will be restored rapidly to ferrous hemoglobin by the red blood cells without any toxic effects. In our case, the amount of H_2_O_2_ is low enough (≤20 *μ*M for direct treatment and ≤ 400 *μ*M for indirect treatment; Figure 2(b)) to be scavenged by the red blood cells, before large amounts of ferryl hemoglobin will be formed [38]. For NO_2_^−^, a similar oxidation process can happen with the formation of ferric hemoglobin (HbFe^3+^), instead of ferryl hemoglobin [39]. Taken together, it is clear that evaluation of the concentration of NTP-generated ROS/RNS in clinical substrates and the by-products they produce is vital for the safe translation of this technology.

Both direct and indirect NTP modalities have translational potential for the clinic, though they work through different mechanisms due to the different species they deliver. A current limitation of direct NTP is the requirement of direct access to the target, thus restricting this NTP modality to superficial wounds and cancers (e.g., melanoma, head and neck carcinoma) or during surgery. Indirect NTP treatment is one way to circumvent this limitation, and solutions enriched with ROS/RNS by NTP have been delivered to deeper layers of the body for treatment [40–42]. It is essential to highlight that with indirect NTP treatment, only the persistent ROS/RNS are delivered to deep tissue, because ROS/RNS with shorter lifetimes undergo reactions to form more stable species in the liquid. Based on our results in this study, it appears that intravenous delivery of NTP-enriched solutions for therapy may be difficult, as these species are lost to the blood in-transit. Specifically, ONOO^−^ was undetectable in under 60 seconds (Figure 4(a)), while H_2_O_2_ was undetectable in under 30 seconds (Figure 2(f)). Total RNS was still detectable at 60 seconds, but at very low levels (Figure 2(e)). However, considering the location of the injection site to the target and the stability of the ROS/RNS species, indirect NTP treatment could be optimized for clinical application. In addition, other indirect NTP delivery methods (e.g., local application, perfusion in body cavities) could also provide promising options. From the data presented herein, it is clear that the clinical benefit of ROS/RNS generated by indirect NTP needs to be thoroughly investigated. A comparison with chemically synthesized ROS/RNS is also required to determine if indirect NTP provides additional advantages. These fundamental studies must be application-specific (e.g., wound healing, cancer therapy) and concentration-specific due to the pleiotropic effects of ROS/RNS [43].

In contrast to indirect NTP, during direct NTP treatment of the target substrate, a unique mixture of short-lived chemical species (e.g., •OH, O, and •NO) can be delivered along with the persistent ROS/RNS (e.g., NO_2_^−^, H_2_O_2_, and ONOO^−^) to induce a biological response [21]. It is important to note that while short-lived species were not measured in this study, their therapeutic effects cannot be discounted in direct NTP treatment, due to the close proximity of the generation site to the target. Recently, the importance of these short-lived species for direct NTP therapy has surfaced, which is in agreement with current understanding in redox medicine [25, 43, 44]. While direct treatment is also known to generate UV radiation, several reports have shown that the amount generated by DBDs does not contribute to the overall biological effect [45, 46]. As it stands, direct NTP treatment is a valuable method of controllably generating and delivering these short-lived ROS/RNS in the clinical setting, which requires further investigation in blood and other biological solutions.

## 5. Conclusions

In summary, we have evaluated the stability of persistent ROS/RNS (i.e., NO_2_^−^, H_2_O_2_, and ONOO^−^) in clinically relevant solutions following direct and indirect NTP treatment. A luminescent Eu(III) probe was able to rapidly and selectively detect ONOO^−^ not only in PBS and BP but also in processed whole blood. NTP-generated ONOO^−^ was detected in all three liquids for up to at least 30 seconds, thus highlighting its therapeutic potential. Treatment of different components of the blood revealed that mainly the cellular and platelet components of blood are responsible for scavenging RNS. On the other hand, both BP and the other blood components have potent scavenging potential against H_2_O_2_ and ONOO^−^. Taken together, our results provide fundamental insight into NTP interactions with blood and suggest that intravenous delivery of NTP-enriched solutions for therapy may be difficult, as a variety of reactive species may be lost to the blood in-transit.

By considering, collectively, the site for introducing NTP-generated ROS/RNS, the stability of the therapeutic species, and the distance to the biological target site, more informed decisions on clinical application of direct and indirect NTP therapy can be made. In the field of plasma medicine, this has high translational importance for NTP therapy in diverse clinical contexts and applications.

## Data Availability

The datasets used and/or analyzed during the current study are available from the corresponding authors on reasonable request.

## Supplementary Materials

Supplementary Materials The file includes *analysis* on the selectivity of the nitrate/nitrite fluorometric assay kit that was used in our study details on peroxynitrite measurements and calibration curve.
