# Supplementary material for: Critical Evaluation of the Interaction of Reactive Oxygen and Nitrogen Species with Blood to Inform the Clinical Translation of Nonthermal Plasma Therapy
Source: Oxid Med Cell Longev. 2020 Dec 3;2020:9750206. doi: 10.1155/2020/9750206 (PMC7728471; doi:10.1155/2020/9750206)
Supplement: Supplementary Materials — The file includes analysis on the selectivity of the nitrate/nitrite fluorometric assay kit that was used in our study details on peroxynitrite measurements and calibration curve. [file 9750206.f1.docx]

**Supplementary Materials**

**Title:** Critical evaluation of the interaction of reactive oxygen and nitrogen species with blood to inform the clinical translation of non-thermal plasma therapy

**Authors:** Abraham Lin^1,2#*^, Eline Biscop^1,2#^, Colum Breen^3^, Stephen J. Butler^3*^, Evelien Smits^2,4^, Annemie Bogaerts^1^

**Affiliations:** ^1^PLASMANT-Research Group; University of Antwerp; 2601, Antwerpen-Wilrijk, Belgium

^2^Center for Oncological Research―Integrated Personalized & Precision Oncology Network (IPPON); University of Antwerp; 2601, Antwerpen-Wilrijk, Belgium

^3^Department of Chemistry; Loughborough University; LE11 3TU Loughborough, United Kingdom

^4^Center for Cell Therapy and Regenerative Medicine; Antwerp University Hospital; 2650, Antwerp-Edegem, Belgium

*** Corresponding Author:** Abraham.Lin@uantwerpen.be

* Co-corresponding author: S.J.Butler@lboro.ac.uk; As the principle investigator of the group that developed the luminescent europium(III) peroxynitrite probe used here, he is the corresponding author with regard to this aspect of the manuscript and the point of contact with regard to future interest in its use .

^#^ Both authors contributed equally and share first authorship

**1. Selectivity of Nitrate/Nitrite Fluorometric Assay Kit**

The selectivity of the Nitrate/Nitrite Fluorometric Assay Kit (780051, Cayman Chemical) used in this study was also evaluated. According to the literature and the manufacturer, the detection of NO_2_^–^ is based on the reaction with 2,3-diaminonapthalene (DAN), followed by sodium hydroxide (NaOH), to form the fluorescent product 1(H)-napthotriazole. However, since NTP is also known to generate ONOO^–^, which is molecularly similar to NO_2_^–^, it is crucial to test its reactivity to DAN to form the fluorescent product. The addition of a fixed concentration of ONOO^–^ (18 µM) to DAN with increasing concentrations of NO_2_^–^ (0-10 µM) resulted in higher fluorescent signals (**Fig. S1**). A linear response to the concentration of the NO_2_^-^ and ONOO mixture indicated that this assay is not specific for NO_2_^–^ (or NO_3_^–^) as the name of the kit would suggest. Therefore, the results obtained from using this assay kit reflect the collective RNS concentration following NTP treatment.

Overlooking this potential interference would lead to misinterpretation and oversimplification of results in complex cellular/biological systems. In biological systems, this assay is often used to determine nitric oxide concentrations, as the assay states that NO_2_^–^ and NO_3_^–^ are the stable products of nitric oxide. However, intracellularly, nitric oxide could also react with superoxide and produce ONOO^–^. As we show here, ONOO^–^ reacts with DAN to produce the fluorescence signal, much higher than shown in the calibration curve using pure NO_2_^–^. While intracellularly generated ONOO^–^ is likely to react with other biological molecules (e.g. amino acids, heme centers), its influence on the assay should be checked and accounted for. In the context of NTP systems, which generate a mixture of RNS, it is clear that this assay can only be used to measure the total amount of RNS, and insight into NO_2_^–^ cannot be delineated without the use of specific ONOO^–^ scavengers or more specie-specific probes. Here, in our study, we used a specific ONOO^–^ luminescent probe (**Eu.1**) to investigate direct NTP-generated ONOO^–^ [28].


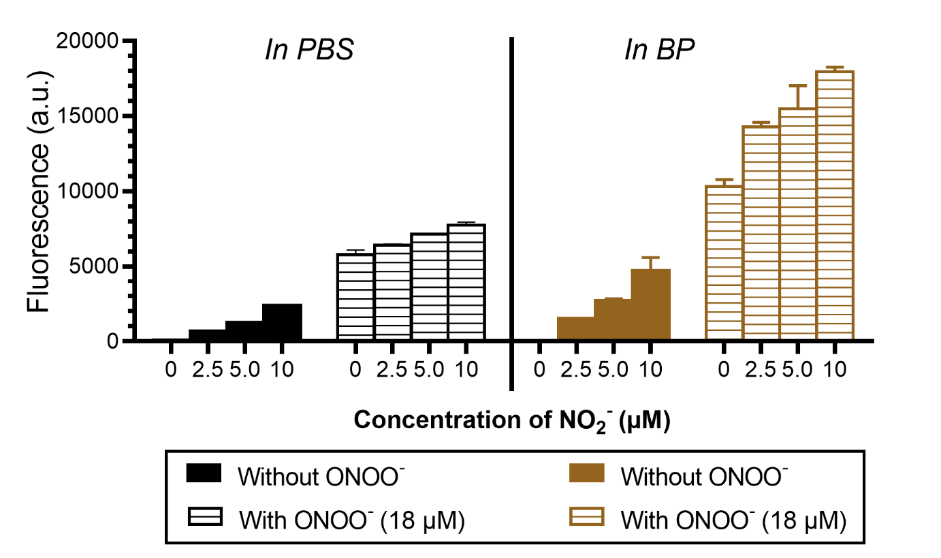
**Figure S1.** The selectivity of the Nitrate/Nitrite Fluorometric Assay Kit (780051, Cayman Chemical) used in this study was determined. The fluorescence signal of the product at increasing NO_2_^–^ concentrations (0-10 µM), with and without the addition of ONOO^–^ (18 µM), was measured in PBS (left) and BP (right). Data are represented as mean± SEM.

**2. ONOO^–^ measurements**

*2.1 Plate reader*

96 black well plates were used for plate reader-based analysis. For calibration data the plate was read using time-resolved emission (BMG Clariostar, λ_ex_ = 292 – 366 nm (TR ex filter), λ_em_ = 605 – 630 nm, int. time = 60 – 400 µs). All measurements were completed in triplicate. For NTP-generated ONOO^–^, measurements were performed at the University of Antwerp using time-resolved emission (λ_ex_ = 340 nm, λ_­em_ = 615 ± 10 nm, int. time = 60 – 400 µs).

*2.2 General procedure for calibration plots*

A known concentration stock solution of ONOO^–^ was prepared in 0.1 M NaOH and kept at 0°C for the duration of the experiment. A known volume was added to 100 µL of Eu.1 (100 µM) in the relevant solvent (PBS or BP). The concentration of ONOO^–^ was increased to a total concentration of 200 µM. Total volume addition did not surpass 5 µL. Emission intensity was measured using conditions as described in Section 1.1.


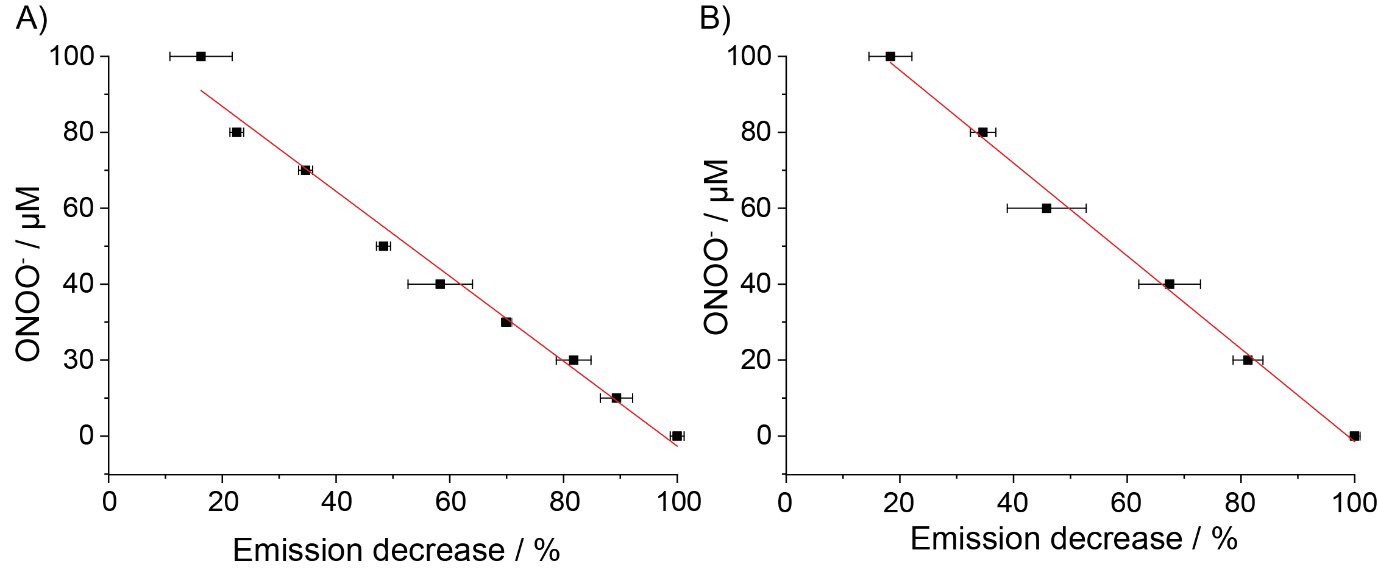
**Figure S2.** Calibration standards for Eu.1 (100 µM) in A) 100 mM PBS, pH 7.4, and in B) human BP. Conditions: λ_ex_ = 292 – 366 nm (TR ex filter), λ_em_ = 605 – 630 nm, int. time = 60 – 400 µs. Calibration standards were generated at Loughborough University and confirmed at University of Antwerp.
